# Enhanced antimicrobial peptide-induced activity in the mollusc Toll-2 family through evolution via tandem Toll/interleukin-1 receptor

**DOI:** 10.1098/rsos.160123

**Published:** 2016-06-15

**Authors:** Jun Cao, Yihong Chen, Min Jin, Qian Ren

**Affiliations:** 1 Jiangsu Key Laboratory for Biodiversity and Biotechnology and Jiangsu Key Laboratory for Aquatic Crustacean Diseases, College of Life Sciences, Nanjing Normal University, Nanjing 210046, People's Republic of China; 2 Institute of Life Sciences, Jiangsu University, Zhenjiang, Jiangsu, People's Republic of China; 3 State Key Laboratory Breeding Base of Marine Genetic Resource, Third Institute of Oceanography, SOA, Xiamen 361005, People's Republic of China; 4 MOE Key Laboratory of Aquatic Product Safety/State Key Laboratory of Biocontrol, School of Marine Sciences, Sun Yat-sen University, Guangzhou, People's Republic of China

**Keywords:** Toll, tandem Toll/interleukin-1 receptor, evolution, over expression, mollusc

## Abstract

Toll receptors play an important role in the innate immunity of invertebrates. All reported Tolls have only one Toll/interleukin-1 receptor (TIR) domain at the C-terminal. In this study, numerous Tolls with tandem TIRs at the C-terminal were found in molluscs. Such Tolls presented an extra TIR (TIR-1) compared with Toll-I. Thus, Toll-I might be the ancestor of tandem TIRs containing Toll. To test this hypothesis, 83 *Toll-I* and *Toll-2* (most have two TIRs, but others seem to be the evolutionary intermediates) genes from 29 shellfish species were identified. These Tolls were divided into nine groups based on phylogenetic analyses. A strong correlation between phylogeny and motif composition was found. All Toll proteins contained the TIR-2 domain, whereas the TIR-1 domain only existed in some Toll-2 protein, suggesting that TIR-1 domain insertion may play an important role in Toll protein evolution. Further analyses of functional divergence and adaptive evolution showed that some of the critical sites responsible for functional divergence may have been under positive selection. An additional intragenic recombination played an important role in the evolution of the *Toll-I* and *Toll-2* genes. To investigate the functional difference of Toll-I and Toll-2, over expression of Hcu_Toll-I or Hcu_Toll-2-2 in *Drosophila* S2 cells was performed. Results showed that Hcu_Toll-2-2 had stronger antimicrobial peptide (AMP) activity than Hcu_Toll-I. Therefore, enhanced AMP-induced activity resulted from tandem TIRs in Toll-2s of molluscs during evolution history.

## 1. Introduction

Invertebrate species account for 95% of the total number of animals in the world [1]. Similar to vertebrates, they suffer from pathogens, such as microbes and viruses. For survival, invertebrates have developed the ability to resist pathogens during evolution. Invertebrates may lack an adaptive immunity system as that in vertebrates, but they possess a powerful and high-performance innate immunity system [2].

Among the signalling pathways of the invertebrate immune system, the Toll signalling pathway is the best characterized one. The *Toll* gene was originally identified with function for specifying dorsal–ventral polarity of the *Drosophila* embryo [3]. Tolls are ubiquitous in embryos, but they are only activated by spatially restricted cleavage by Spätzle (Spz) [4]. A previous study reported that the *Drosophila* Toll pathway shows remarkable similarity to the mammalian interleukin-1 pathway, which activates NF-κB, a protein responsible for inflammatory and immune responses [5]. Toll-9 has been proven to play a role in fungal and Gram-positive bacterial defence of *Drosophila*, independent of its morphogenetic functions [4,6]. The discovery of immune function for Toll in flies has led to the identification of vertebrate Toll-like receptors (TLRs) [7]. TLRs have been identified in animals ranging from cnidarians to mammals.

Pathogen-associated molecular patterns (PAMPs) are small molecular motifs conserved within groups of pathogens. Upon pathogen invasion, they are recognized by TLRs and other pattern recognition receptors (PRRs) and initiate immune response. In *Drosophila*, a cascade reaction system is triggered once PRRs recognize PAMPs. This system contains Spz, myeloid differentiation factor 88 (MyD88), tube, pelle, dorsal, inhibitor of NF-κB kinases (IKKs) and cactus. This cascade activates NF-κB, which enhances the expression of immune factor genes, such as *AMPs*, *lysozyme* and *anti-lipopolysaccharides* (*ALFs*) for resistance to infection. The *Drosophila* genome encodes nine Toll proteins, but only Toll-9 has a role in immunity. Some vertebrate species have more than one TLR involved in immune responses; for example, *Homo sapiens* TLR1, TLR2, TLR3, TLR4, TLR5, TLR6, TLR7 and TLR9 are all engaged in the recognition of different PAMPs and activate distinct immune responses.

To date, TLRs play a role in infection prevention and other immune-related processes. A previous study found that dual antigen-specific B cell receptor (BCR) and TLR engagement can fine-tune functional B cell responses, directly linking cell-intrinsic innate and adaptive immune programmes [8]. TLR activation upregulates pro-tumourigenic pathways, including the induction of inducible nitric oxide synthase (iNOS2) and cyclooxygenase (COX) 2, to promote a feed-forward loop leading to tumour progression and the development of more aggressive tumour phenotypes [9]. Furthermore, TLR4 is expressed in intestinal stem cells and regulates their proliferation and apoptosis via the p53 upregulated modulator of apoptosis [10]. Notably, an increasing number of studies have indicated that TLRs are engaged in autoimmunization; for example, TLR4 in T cells promotes autoimmune inflammation; TLR7, TLR8 and TLR9 play a key role in promoting the production of autoantibodies reactive with DNA- or RNA-associated autoantigens [11,12]. Functions of TLRs in invertebrates other than *Drosophila* have also been investigated in recent years, but most of these studies focused on their roles in anti-pathogens [13,14].

The reason for the functional diversity in TLRs is implied in their structures. Toll receptors are transmembrane proteins with extracellular leucine-rich repeat (LRR) motifs and the Toll/interleukin-1 receptor (TIR) domain [15]. The ectodomain of TLRs contains LRR motifs, which are defined by a consensus sequence of 24–29 amino acids in length, and the number of LRRs in different TLRs varies from 19 to 26 [16]. Such tandem arrays of LRRs have been found in proteins involved in ribosome and DNA binding, signal transduction, enzyme inhibition and cell adhesion [17]. The sequences of the TLR intracellular domain have similarities to the mammalian interleukin-1 receptors, called the TIR domain. This domain is composed of about 150 amino acid residues. Recognition of PAMPs and cytokines by TLRs leads to the stabilization of a dimeric form of the receptor through TIR domains, as well as providing a scaffold or recruitment of cytosolic adaptor proteins [18]. To date, most TLRs that have been investigated only contain one TIR domain.

In our previous study, a Toll receptor (Hcu_Toll-2-1) with tandem TIR domains at the C-terminal was found and studied in *Hyriopsis cumingii* [19]. A single TIR-containing Toll (Hcu_Toll-I) sharing similarities to Toll-I from *Mytilus galloprovincialis* (AFU48617.1) was also identified from *H. cumingii.* Hcu_Toll-2-1 and Hcu_Toll-I have similar domain structures but an extra TIR (TIR-1) is found in Hcu_Toll-2-1. Thus, *Hcu_Toll-I* may be the ancestor gene of *Hcu_Toll-2-1*. To test this hypothesis, transcriptome sequencing was performed in 29 mollusc species to obtain more sequences similar to the *Toll-I* or *Hcu_Toll-2-1* genes.

A total of 83 *Toll-I* and *Toll-2* genes from 29 aquatic molluscs were identified. Phylogenetic analyses revealed that these Tolls could be divided into the Toll-I and Toll-2 families. Most members of Group VII in the Toll-2 family contained two TIR domains. Functional divergence analyses also indicated that the *Toll* genes diverged functionally from each other, causing different evolutionary rates. Over expression of Hcu_Toll-2-2 or Hcu_Toll-I in *Drosophila* S2 cells showed that tandem TIR-containing Toll (Hcu_Toll-2-2) had stronger antimicrobial peptide (AMP)-induced activity than single TIR Toll (Hcu_Toll-I). Therefore, tandem TIR Toll genes have been preserved in mollusc species during evolution, which possibly resulted from its enhanced function.

## 2. Material and methods

### 2.1. Animals

In this study, 29 mollusc species (species tree is shown in the electronic supplementary material, S1) were selected to investigate the evolution of mollusc Toll receptors. Three freshwater mussels, namely, *H. cumingii*, *Sinanodonta woodiana* and *Cristaria plicata*, were purchased from Wuhu City, Anhui Province, China. Another 26 seawater mollusc species were purchased from Nanjing and Hangzhou aquatic markets. Among these 26 species, *Peronidia zyonoensis*, *Qicaibei*, *Wenbei* and *Hongbei* were purchased from Hangzhou City, Zhejiang Province, China. In addition to these four species, the remaining species were purchased from Nanjing, Jiangsu Province, China. Among these 29 species, only six species (i.e. *Haliotis rubra*, *Neptunea cumingi*, *Cymbium melo*, *Babylonia areolata*, *Rapana bezona* and *Turritella terebra*) belong to Gastropoda. Other species belong to Bivalvia. Detailed information of species in this study can be found in the electronic supplementary material, S2. Given that no Latin name could be obtained, the Chinese Pinyin of *Qicaibei, Wenbei*, *Hongbei* and *Jinqianbei* were used in this study.

### 2.2. Transcriptome sequencing of mollusc species and cDNA cloning

The transcriptomes of mollusc species were sequenced using Illumina HiSeq™ 2000. The raw reads were de novo assembled using Trinity program. The transcriptome assemblies were searched for sequences similar to *Toll-I* or *Hcu_Toll-2-1* using the tBLASTn algorithm. BLAST results showed that 83 *Toll-I* and *Toll-2* genes were found. Some of them possessed complete coding regions. However, some of them had no complete coding region and only had 3′ or 5′-ends. To obtain the coding region of these sequences, 3′ or 5′ RACE methods were employed using the SMARTer RACE 5′/3′ Kit (Clontech), following the manufacturer's manual. Detailed methods can be found in our previously published paper [19]. The primers used for RACE are shown in the electronic supplementary material, S3.

### 2.3. Phylogenetic analyses of the Toll-I and Toll-2 protein families

Multiple sequence alignments of the TIR-2 domain sequences were performed using MUSCLE 3.52, followed by manual comparisons and refinement [20]. Phylogenetic analyses of the Toll protein family, based on TIR-2 domain sequences, were performed with the neighbour-joining (NJ) method using MEGA 6 [21]. We also used TLR1 protein from *Mus musculus* as an outgroup. Bootstrap support values were estimated using 1000 pseudo-replicates.

### 2.4. Functional divergence analyses

Some residues are highly conserved and others are highly variable in evolution. We used DIVERGE (v. 2.0) [22,23] to analyse the type I functional divergence between different groups of Toll receptor proteins. The functional divergence between two groups was measured as the coefficient of functional divergence (*θ*). A coefficient equal to 0 indicates that the evolutionary rate of the duplicate genes at each site is entirely consistent. When the coefficient is greater than 0, the evolutionary rate of the duplicate genes at some critical amino acid residues is different. The software will predict these sites responding for functional divergence.

### 2.5. Site-specific selection assessment and testing

We used the Selecton Server (http://selecton.tau.ac.il/) [24] to calculate site-specific purifying and positive selection. In this study, *K*_a_*/K*_s_ values were used to estimate two types of substitution events by calculating the synonymous rate (*K*_s_) and the non-synonymous rate (*K*_a_) at each codon. Three evolutionary models (M8 (*ω_s_* ≥ 1), M7 (beta) and M5 (gamma)) were used to describe, in probabilistic terms, how the characters evolve. Each of the models used different biological assumptions and the model that best fit the data was selected. These models all assumed a statistical distribution to account for heterogeneous *K*_a_*/K*_s_ values among sites. The distributions were approximated using eight discrete categories and the *K*_a_*/K*_s_ values were computed by calculating the expectation of a posterior distribution [24].

### 2.6. Detection of recombination events

Coding sequence (CDS) in different Toll groups was first aligned. The recombination detection program RDP v. 3.44 [25] was used to explore potential recombination events between divergent nucleotide sequences. This software embeds different methods for detecting recombination signals. In this study, three methods (RDP [26], Geneconv [27] and MaxChi [28]) were used to detect signals. The highest acceptable *P* cut-off value was set to 0.05. Significance was evaluated with 100 permutation tests.

### 2.7. Construction of recombinant plasmid

The nucleotide sequences corresponding to the amino acid sequences of LRR + TM + TIR-1, LRR + TM + TIR-1 + TIR-2 of *Hcu_Toll-2-2* and LRR + TM + TIR of *Hcu_Toll-I* were amplified using the primers in the electronic supplementary material, S3 (Pac-*Hcu_Toll-2-2*-F, Pac-*Hcu_Toll-2-2-*R1, Pac-*Hcu_Toll-2-2-*R2; Pac-*Hcu_Toll-I-*F, Pac-*Hcu_Toll-I-*R). The amplified fragments were digested with Kpn I and Apa I and then ligated into a pAc5.1/V5–His B vector (Invitrogen, USA). The recombinant plasmids were sequenced to ensure correct insertion. To construct the recombinant plasmid of LRR + TM + TIR-2, in-fusion technology was used. In detail, two pairs of primers (*Hcu_Toll-2-2*-LRR-F: 5′-CCCGGATCGGGGTACCATGATAAGCGGACTGCAGCATTTAG-3′, *Hcu_Toll-2-2*-LRR-R: 5′-CTCGTCTTTATTTGCCACGTCTTTG-3′; *Hcu_Toll-2-2*-TIR-2-F: 5′-GCAAATAAAGACGAGAGGATCC ACGCTTTCGTTTC-3′, *Hcu_Toll-2-2*-TIR-2-R: 5′-TTCGAACCGCGGGCCCTCTGTGATCTCTATCGCAACATC-3′) were used to amplify the *LRR* + *TM* and *TIR-2* cDNA sequences, respectively. The 16 bp vector-specific sequences added on the 5′-ends of *Hcu_Toll-2-2*-LRR-F and *Hcu_Toll-2-2*-TIR-2-R were underlined. Moreover, a 15 bp sequence of *Hcu_Toll-2-2*-*TIR-2*-F reverse complementary to the first 15 bp of *Hcu_Toll-2-2*-*LRR*-R is underlined with dots. The two purified fragments were then ligated into the linearized pAc5.1/V5–His B vector (double digestion with Kpn I and Apa I). About 1 µl of linearized pAc5.1/V5–His B vector, 3 µl of fragment 1, 3 µl of fragment 2 and 2 µl of In-Fusion HD Master Mix were combined and incubated for 15 min at 50°C. The recombinant plasmid was finally transformed into Trans1-T1 chemically competent cells. The recombinant plasmid was also sequenced to ensure correct insertion.

### 2.8. Dual-luciferase activity assay in S2 cells

Dual-luciferase activity assay in S2 cells was conducted according to the protocol of a previously published paper. In brief, the abovementioned recombinant plasmid or empty pAc5.1/V5–His B plasmid (0.3 µg) along with pGL-Pen4 or empty pGL3-Basic plasmid (0.2 µg) and 0.02 µg of pRL-TK plasmid (wild-type *Renilla* luciferase control reporter vector; Promega, USA) were co-transfected into *Drosophila* S2 cells. The reporter gene plasmid (pGL-*Pen4*) was constructed using the promoter sequence of shrimp Penaeidin-4 (PEN4). S2 cells were cultured in standard *Drosophila* medium (serum-free; Invitrogen, USA) containing 10% fetal bovine serum (Invitrogen) at 27°C. Cellfectin II reagent (Invitrogen) was used for DNA transfection into S2 cells. The firefly and *Renilla* luciferase activities were measured after 48 h of transfection with the Dual-Luciferase Reporter Assay System (Promega), according to the manufacturer's instructions. All these assays were performed from three independent transfections.

## 3. Results and discussion

### 3.1. Transcriptome sequencing and identification of *Toll-2* and *Toll-I*

A total of 83 *Toll-I* and *Toll-2* (most have two TIRs) genes from 29 mollusc species were identified using high-throughput sequencing and RACE technology (electronic supplementary material, S1). Based on their living environment, these mollusc species could be divided into freshwater and seawater species. Among these species, only *H. cumingii*, *S. woodiana* and *C. plicata* are freshwater mussels. According to the classification of species, these species could be divided into Gastropoda and Bivalvia. Among these species, *H. rubra*, *N. cumingi*, *C. melo*, *B. areolata*, *R. bezona* and *T. terebra* belong to Gastropoda. In some species, only *Toll-I* could be found and no *Toll-2* could be identified. One *Toll-I* in *Pinna rudis*, *C. melo*, *Crassostrea gigas*, *Hongbei* and *Scapharca subcrenata*, two *Toll-I* in *Tegillarca granosa* and three *Toll-I* in *Moerella iridescens*, *Mytilus edulis* and *Mactra veneriformis* were identified. In *N. cumingi*, only one *Toll-2* was found and no *Toll-I* could be identified. In other species, both *Toll-I* and *Toll-2* could be found. Based on their isoform number, they could be divided into nine different situations. (i) Only one *Toll-I* and one *Toll-2* were identified in *H. rubra*, *R. bezona*, *Ruditapes philippinarum*, *B. areolata*, *T. terebra*, *Saxidomus purpuratus*, *Sinonovacula constricta*, *Qicaibei* and *Cyclina sinensis*. (ii) In *H. cumingii*, one *Toll-I* and two *Toll-2* were found. (iii) In *Meretrix meretrix*, only one *Toll-I* but as many as six *Toll-2* were identified. (iv) In *Solen strictus* and *P. zyonoensis*, two *Toll-I* and one *Toll-2* were identified. (v) Two *Toll-I* and two *Toll-2* were identified from *Panopea abrupta* and *Chlamys farreri*, respectively. (vi) In *Wenbei*, two *Toll-I* and three *Toll-2* were identified. (vii) In *C. plicata*, two *Toll-I* and five *Toll-2* were found. (viii) In *Jinqianbei*, three *Toll-I* and three *Toll-2* were identified. (ix) Four *Toll-I* and two *Toll-2* were identified from *S. woodiana*.

### 3.2. Phylogenetic and structural analyses of the Toll-I and Toll-2 proteins

To predict the evolutionary relationships of the Toll-I and Toll-2 families, we constructed a NJ tree based on alignment of the TIR-2 sequences of the Toll-I and Toll-2 proteins. The majority of the phylogenetic clades had well-supported bootstrap values. These genes were categorized into nine major groups, named Groups I to IX. Groups I, II, III, IV, V and VI belong to the *Toll-I* gene family, whereas Groups VII, VIII and IX belong to the *Toll-2* gene family. The largest group was VII, which contained 24 members. Group VI contained only four genes.

**Figure 1.**
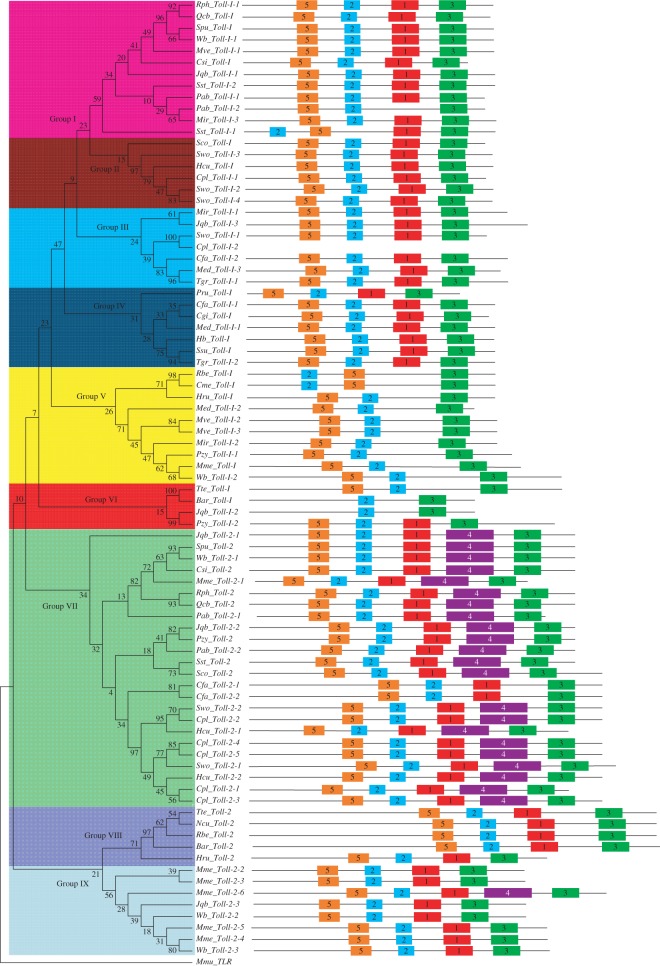
Phylogenetic relationships and structure analyses of the Toll-I and Toll-2 protein family. The numbers above branches show bootstrap values from NJ. All motifs were identified by MEME using the complete amino acid sequence of the Toll-I and Toll-2 family. Details of the motifs refer to the electronic supplementary material, S4.

To further confirm the phylogenetic relationships and to examine the diversity of Toll proteins, we further searched for conserved motifs in Toll proteins using the MEME web server (http://meme.sdsc.edu) [29]. As shown in figure 1, five conserved motifs (motifs 1–5) were identified in these Toll proteins. All the predicted Tolls had conserved motifs 2 and 3, and the genes in the same group had similar conserved motifs, but some divergence was observed between groups. These results suggested that the Toll genes in the same group may have similar functions and some specific motif architectures may have important effects on group-specific functions. We also noticed that most proteins in Group VII contained motif 4, which did not exist in other groups, except one member (Mme_Toll-2-6) in Group IX, implying that this motif may be related to the specific functions of these proteins. Therefore, motif compositions of the Toll proteins in each group may provide additional support for phylogenetic analyses. We also used Pfam [30] to identify major domains of the Toll protein family. Interestingly, we also found that most members of Group VII possessed two TIR domains (one was TIR-1 (motif 4) and the other was TIR-2 (motif 3)), whereas other Tolls only contained the TIR-2 domain. Among these Toll-2 members, 14 Toll-2s were the evolutionary intermediates between single TIR Tolls and tandem TIR Tolls. Among these 14 Tolls, five Tolls were from Gastropoda. In Gastropoda, no complete tandem TIR Tolls could be found. BLASTP results showed that these 14 Tolls had similarities to Hcu_Toll-2-1, which contained two TIRs. However, SMART analysis showed that these 14 Tolls contained incomplete tandem TTRs. Thus, these 14 Tolls may be evolutionary intermediates.

Domain insertion is usually thought to evolve through recombination events and intragenic duplication [31]. The creation of new multi-domain architectures is an important mechanism that provides opportunities for the organism to expand its repertoire of cellular functions, such as transcriptional regulation, protein transport and assembly [32–34]. Furthermore, protein domain insertion may constitute a source of variability. In the human genome, duplications are more common in genes containing repeated domains than in non-repeated ones [31]. Domain insertion is quite important in evolution, as it provides a path where proteins can evolve by removing or adding functionally similar or distinct blocks [35]. In the current study, we identified some multi-TIR domains in Tolls. The presence of two TIR domains contributed to the complexity of this gene family. Its influence on the function of Toll proteins remains to be examined. However, our findings suggested that TIR-1 domain insertion in most members of Group VII may play an important role in Toll protein evolution.

### 3.3. Functional divergence analyses of the Toll proteins

To further study whether amino acid substitutions cause adaptive functional diversification, we used the program DIVERGE [22,23] to estimate type-I functional divergence between different Toll groups. We compared 36 pairs of paralogous members and estimated the evolutionary rate of each amino acid site. Our results indicated that the coefficient of functional divergence (*θ*) values among these pairs was less than 1 (table 1), indicating significant site-specific alteration in selective constraints of most members of the Toll group pairs. We also predicted a few critical residues associated with functional divergence. For example, over 90 critical sites were predicted in the II/VI, II/V, II/VIII, II/IX, III/VII, III/IX, IV/IX, V/VII, V/VIII and V/IX pairs, whereas no site was predicted in the I/VI, II/III, II/VI, III/IV, III/VI, V/VI, VI/VII, VI/VIII, VI/IX and VIII/IX pairs when the cut-off value was 0.5. The highest theta values (*θ*) were observed in the II/IV, II/IX, III/IX and V/VIII pairs (table 1), suggesting a higher evolutionary rate between them. Thus, given the different evolutionary rates, the *Toll* genes diverged functionally from each other. The amino acid mutations promoted the functional evolution and divergence of the *Toll* genes as an adaptation of the species to the changing environment.

**Table 1.** Functional divergence estimated between different groups of Toll proteins. Theta, coefficient of functional divergence; s.e., standard error; LRT, likelihood ratio test.

comparison	theta	s.e.	LRT	*N*(0.5)^a^	*N*(0.7)^a^
I/II	0.563707	0.269664	1.237648	3	0
I/III	−0.29163	0.193027	2.38981	2	0
I/IV	0.188822	0.098201	1.263873	1	0
I/V	0.31064	0.101114	11.73627	15	5
I/VI	−1	−1	−1	0	0
I/VII	0.705547	0.07885	77.0213	88	41
I/VIII	0.625755	0.12558	25.13543	85	23
I/XI	0.651601	0.108299	31.51791	77	28
II/III	0.371238	0.713586	0.277641	0	0
II/IV	0.942475	0.303975	8.657418	100	100
II/V	0.754356	0.395619	2.061301	93	4
II/VI	−1	−1	−1	0	0
II/VII	0.626231	0.18053	10.35031	87	11
II/VIII	0.751889	0.304608	5.724468	99	92
II/IX	0.881859	0.325205	8.031021	100	100
III/IV	−1.0643	−23.7985	−1	2	2
III/V	−0.97564	−21.816	−1	3	3
III/VI	−1	−1	−1	0	0
III/VII	−0.03946	0.137657	23.60464	92	35
III/VIII	0.166247	0.241993	4.964715	87	11
III/IX	1.202301	0.23089	21.81064	100	100
IV/V	0.208136	0.101033	4.173458	5	1
IV/VI	−1	−1	−1	0	0
IV/VII	0.575547	0.097513	47.15257	89	40
IV/VIII	0.52178	0.144871	12.76522	41	11
IV/IX	0.756708	0.133149	29.17587	99	64
V/VI	−1	−1	−1	0	0
V/VII	0.560374	0.094396	57.79011	94	48
V/VIII	0.905158	0.157392	30.30209	100	99
V/IX	0.718287	0.132621	28.4354	96	49
VI/VII	−1	−1	−1	0	0
VI/VIII	−1	−1	−1	0	0
VI/IX	−1	−1	−1	0	0
VII/VIII	0.237911	0.095991	14.1618	16	7
VII/IX	0.591538	0.089531	45.39129	78	26
VIII/IX	−1	−1	−1	0	0

^a^*N*(0.5) and *N*(0.7) means the numbers of divergent residues when the cut-off value is 0.5 and 0.7, respectively.

### 3.4. Selective pressure at amino acid sites in the Toll family members

The *K*_a_*/K*_s_ ratio measures selection pressure on amino acid substitutions. A *K*_a_*/K*_s_ ratio greater than 1 suggests positive selection and a ratio less than 1 suggests purifying selection [36]. Amino acids in a protein are usually expected to be under different selective pressures and possess different *K*_a_*/K*_s_ ratios [37]. To test for the presence of positive or negative selection at individual amino acids, the *K*_a_*/K*_s_ ratios were calculated with the Selecton Server (http://selecton.tau.ac.il) [24]. We used three evolutionary models (M8 (*ω*_s_ ≥ 1), M7 (beta), and M5 (gamma)) implemented in this server to perform the tests. The results showed that the *K*_a_*/K*_s_ ratios of the sequences from different Toll groups were significantly different (table 2). For example, higher *K*_a_*/K*_s_ ratios existed in Groups II, VI and IX, indicating a higher evolutionary rate or site-specific selective relaxation within members of the same group. Despite the differences in *K*_a_*/K*_s_ values, all the estimated *K*_a_*/K*_s_ values were substantially lower than 1, suggesting that the Toll sequences within each of the groups were under strong purifying selection pressure. The selection model M7 did not indicate the presence of positively selected sites, whereas the M5 model did in Groups II, V, VI and IX (table 2). These observations suggested that selection spurred the potential for amino acid diversity at some residues, whereas other residues evolved under purifying or neutral selection. These positively selected residues might have changed the protein structure, thereby accelerating functional divergence during long periods of evolution.

**Table 2.** Likelihood values and parameter estimates for different groups of the *Toll-I* and *Toll-2* genes.

groups	selection model	*K*_a_*/K*_s_	log-likelihood	number of positive-selection sites
I	M8 (*ω*_s_ ≥ 1)	0.2496	−23 771.4	0
	M7 (*β*)	0.2353	−23 771.3	0
	M5 (*γ*)	0.2823	−23 800.3	0
II	M8 (*ω*_s_ ≥ 1)	0.4715	−5572.62	95
	M7 (*β*)	0.3714	−5580.1	0
	M5 (*γ*)	0.4949	−5570.53	92
III	M8 (*ω*_s_ ≥ 1)	0.2785	−15 792.5	0
	M7 (*β*)	0.2668	−15 791.2	0
	M5 (*γ*)	0.3354	−15 802.1	0
IV	M8 (*ω*_s_ ≥ 1)	0.2038	−12 771.1	0
	M7 (*β*)	0.1947	−12 768.4	0
	M5 (*γ*)	0.2396	−12 783.1	0
V	M8 (*ω*_s_ ≥ 1)	0.3202	−26 211.9	0
	M7 (*β*)	0.3155	−26 207.7	0
	M5 (*γ*)	0.3843	−26 258.2	2
VI	M8 (*ω*_s_ ≥ 1)	0.3740	−11 774.3	0
	M7 (*β*)	0.3496	−11 772.3	0
	M5 (*γ*)	0.4754	−11 804	8
VII	M8 (*ω*_s_ ≥ 1)	0.2413	−65 514	0
	M7 (*β*)	0.2252	−65 511.6	0
	M5 (*γ*)	0.2638	−65 574.7	0
VIII	M8 (*ω*_s_ ≥ 1)	0.2876	−20 307.7	0
	M7 (*β*)	0.2878	−20 304.4	0
	M5 (*γ*)	0.3206	−20 320.3	0
IX	M8 (*ω*_s_ ≥ 1)	0.3428	−24 351.7	0
	M7 (*β*)	0.3245	−24 358.4	0
	M5 (*γ*)	0.4102	−24 358.4	8

### 3.5. Recombination analysis within *Toll* genes

**Figure 2.**
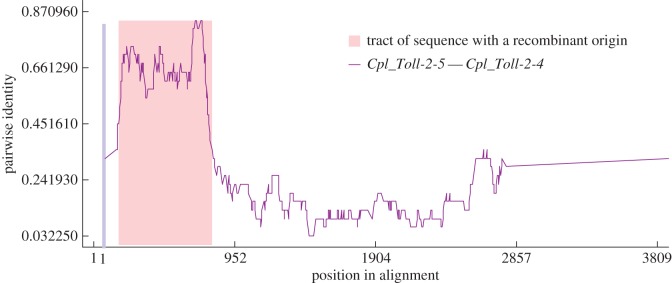
Identification of recombination events between *Cpl_Toll-2-5* and *Cpl_Toll-2-4* genes. The plot display of recombination events was detected by the RDP method.

Recombination plays a key role in the generation of genetic diversity. To determine whether homologous recombination shapes the evolution of the *Toll* genes, we analysed all *Toll* CDS segments to test whether some of them underwent an intragenic recombination event. Recombination signals of the *Toll* genes were investigated with the RDP [26], Geneconv [27] and MaxChi [28] methods embedded in the program RDP v3.44 [25]. All nine groups were found to contain similar mosaic segments, demonstrating that intragenic recombination had occurred. As summarized in table 3, 57 *Toll* genes in these groups exhibited evidence of intragenic recombination (*p* < 0.05 based on 100 permutations). As an example, we presented a recombination event of *Cpl_Toll-2-5* and *Cpl_Toll-2-4* detected by the RDP method (figure 2). A significant recombination event occurred between the 5′-ends of *Cpl_Toll-2-5* and *Cpl_Toll-2-4*. Our results indicated that the *Toll* genes underwent frequent recombination events. Therefore, intragenic recombination played an important role in the evolution of the *Toll* genes, similar to other family genes [38,39]. Further studies are required to evaluate the influence of recombination on function and investigate the mechanisms underlying *Toll* recombination.

**Table 3.** The predicted recombination events for the *Toll-I* and *Toll-2* genes in shellfish.

	recombination methods			
groups	RDP	GENECONV	MaxChi	genes undergone recombination events
I	2	1	6	*Spu_Toll-I*, *Mvw_Toll-I-1*, *Sst_Toll-I-2*, *Mir_Tol-I-3*, *Sco_Toll-I*, *Csi_Toll-I*, *Qcb_Toll-I*, *Pab_Toll-I-1*
II	4	5	3	*Swo_Toll-I-2*, *Swo_Toll-I-3*, *Swo_Toll-I-4*, *Cpl_Toll-I-1*, *Hcu_Toll-I*
III	3	1	3	*Tgr_Toll-I-1*, *Mir_Toll-I-1*, *Swo_Toll-I-1*, *Cfa_Toll-I-2*, *Cpl_Toll-I-2*
IV	3	2	3	*Pru_Toll-I*, *Med_Toll-I-1*, *Cfa_Toll-I-1*
V	8	1	8	*Rbe_Toll-I*, *Hru_Toll-I*, *Cme_Toll-I*, *Mve_Toll-I-2*, *Cme_Toll-I*, *Mve_Toll-I-3*, *Med_Toll-I-2*, *Mir_Toll-I-2*, *Pzy_Toll-I-1*
VI	1	5	5	*Tte_Toll-I*, *Pzy_Toll-I-2*, *Jqb_Toll-I-2*, *Bar_Toll-I*
VII	15	12	24	*Cfa_Toll-2-1*, *Hcu_Toll-2-1*, *Cpl_Toll-2-5*, *Hcu_Toll-2-2*, *Csi_Toll-2*, *Mme_Toll-2-1*, *Cfa_Toll-2-2*, *Cpl_Toll-2-1*, *Cpl_Toll-2-3*, *Swo_Toll-2-1*, *Cpl_Toll-2-4*, *Swo_Toll-2-2*, *Csi_Toll-2*, *Sst_Toll-2*, *Rph_Toll-2*
VIII	3	4	7	*Rbe_Toll-2*, *Ncu_Toll-2*, *Tte_Toll-2*, *Hru_Toll-2*
IX	2	0	3	*Mme_Toll-2-2*, *Wb_Toll-2-3*, *Jqb_Toll-2-3*

### 3.6. Over expression of *Hcu_Toll-2-2* and *Hcu_Toll-I* activates shrimp penaeidin-4

**Figure 3.**
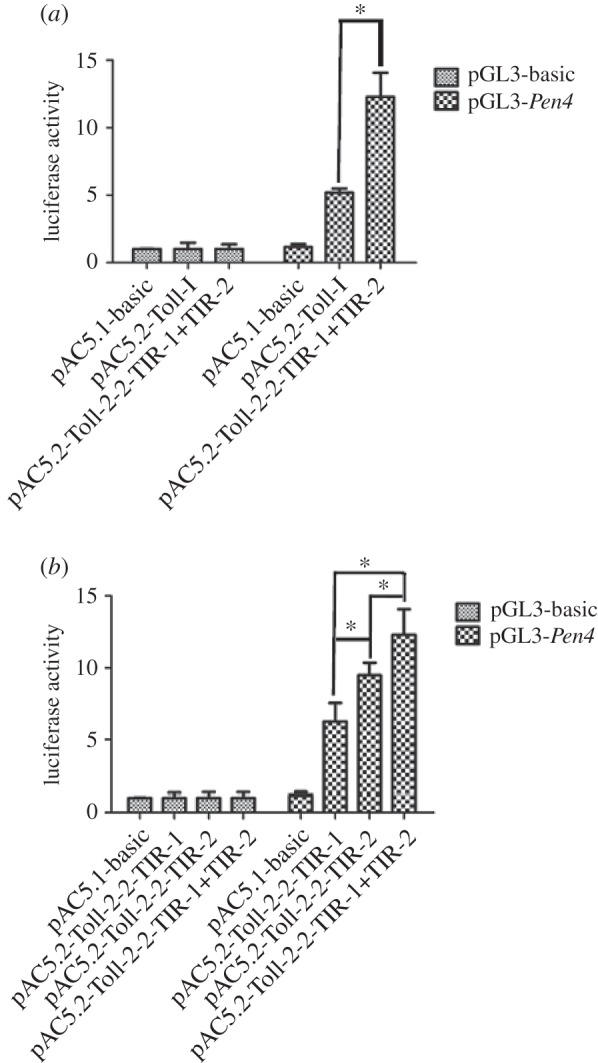
Activation of shrimp *Pen4* antimicrobial peptide gene by over expression of the full length of *Hcu_Toll-I* and *Hcu_Toll-2-2* (*a*), *Hcu_Toll-2-2*-TIR-1, *Hcu_Toll-2-2*-TIR-2 and *Hcu_Toll-2-2*-TIR-1 + TIR-2 (*b*) in S2 cells. The detailed methods could be seen in Material and methods section. All data are representative of three independent experiments. The bars indicate the s.d. of the luciferase activity (*n* = 3).

To analyse the functional differences between tandem TIRs (TIR-1 and TIR-2) containing Toll (Hcu_Toll-2-2) and single TIR domain containing Toll (Hcu_Toll-I), over expression of *Hcu_Toll-2-2* or *Hcu_Toll-I* in *Drosophila* S2 cells was performed. Dual-luciferase reporter assays were performed and both Hcu_Toll-2-2 and Hcu_Toll-I could activate the promoter activity of shrimp *penaeidin*-*4*. However, Hcu_Toll-2-2 had stronger AMP-induced activity than Hcu_Toll-I. The difference in AMP-induced activity of TIR-1 and TIR-2 of Hcu_Toll-2-2 was further investigated. As shown in figure 3*b*, Hcu_Toll-2-2 (containing LRRs + TM + TIR-1 + TIR-2) had stronger AMP-induced activity than Hcu_Toll-2-2-TIR-1 (including LRRs + TM + TIR-1) and Hcu_Toll-2-2-TIR-2 (LRRs + TM + TIR-2). Hcu_Toll-2-2-TIR-2 had stronger AMP-induced activity than Hcu_Toll-2-2-TIR-1 (figure 3).

SMART analysis showed that Hcu_Toll-I had four LRRs, followed by one LRR_CT, two LRRs, one LRR_CT, one TM and one TIR named TIR-2. In terms of Hcu_Toll-2-2, in addition to a TIR (TIR-1), the other was similar to Hcu_Toll-I. Phylogenetic analysis demonstrated that Toll-I was the ancestor of Toll-2. Thus, the enhanced AMP-induced activity of Toll-2 may result from its evolution into tandem TIRs. A previous study confirmed that LRR repeat domains in many plant resistance genes play important roles in plant innate immune systems [40]. Activation of the Toll signalling pathway can induce the interaction of Toll and Myd88 adaptor molecules through its TIR domain. Tandem TIRs of Toll-2 may have more interaction sites and may bind more Myd88 molecules, which induce strong AMP-induced activity. The enhanced AMP-induced activity resulted in tandem TIR-containing Tolls existing in molluscs during evolution.

## Supplementary Material

Supplementary Fig. 1

## Supplementary Material

List of the Toll-I and Toll-2 genes in different species used in this research

## Supplementary Material

Primers used in this study

## Supplementary Material

Supplementary material, S4. Detail of five motifs.
