# Supplementary material for: Enhanced antimicrobial peptide-induced activity in the mollusc Toll-2 family through evolution via tandem Toll/interleukin-1 receptor
Source: R Soc Open Sci. 2016 Jun 15;3(6):160123. doi: 10.1098/rsos.160123 (PMC4929906; doi:10.1098/rsos.160123)
Supplement: Supplementary Fig. 1 [file rsos160123supp1.doc]

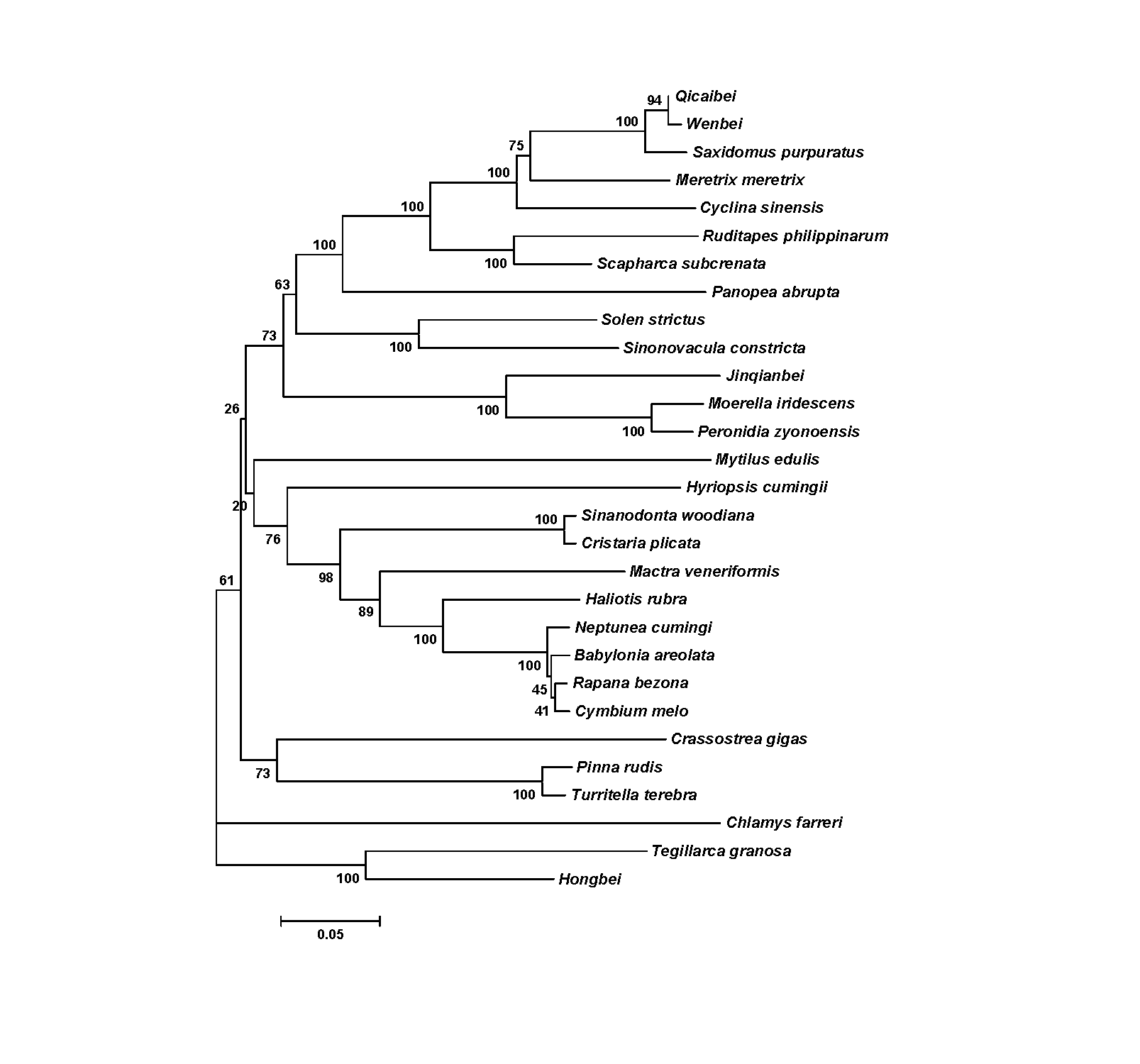


**Supplementary Fig. 1. Phylogenetic relationship of mollusc species.** The phylogenetic tree is reconstructed based on mitochondrial *cytochrome c oxidase subunit I* (*cox1*) protein sequences.
