# Supplementary material for: Enhanced antimicrobial peptide-induced activity in the mollusc Toll-2 family through evolution via tandem Toll/interleukin-1 receptor
Source: R Soc Open Sci. 2016 Jun 15;3(6):160123. doi: 10.1098/rsos.160123 (PMC4929906; doi:10.1098/rsos.160123)
Supplement: List of the Toll-I and Toll-2 genes in different species used in this research [file rsos160123supp2.doc]

**List of the Toll-I and Toll-2 genes in different species used in this research**

| **Species** | **Abbreviation** | **Genes** |
| --- | --- | --- |
| *Mactra veneriformis* | Mve | *Mve_Toll-I-1,Mve_Toll-I-2,Mve_Toll-I-3* |
| *Panopea abrupta* | Pab | *Pab_Toll-1-1,Pab_Toll-1-2,Pab_Toll-2-1,Pab_Toll-2-2* |
| *Haliotis rubra* | Hru | *Hru_Toll-I,Hru_Toll-2* |
| *Sinanodonta woodiana* | Swo | *Swo_Toll-I-1,Swo_Toll-I-2,Swo_Toll-I-3,Swo_Toll-I-4,Swo_Toll-2-1,Swo_Toll-2-2* |
| *Pinna rudis* | Pru | *Pru_Toll-I* |
| *Turritella terebra* | Tte | *Tte_Toll-I,Tte_Toll-2* |
| *Cristaria plicata* | Cpl | *Cpl_Toll-I-1,Cpl_Toll-I-2,Cpl_Toll-2-1,Cpl_Toll-2-2,Cpl_Toll-2-3,Cpl_Toll-2-4,Cpl_Toll-2-5* |
| *Moerella iridescens* | Mir | *Mir_Toll-I-1,Mir_Toll-I-2,Mir_Toll-I-3* |
| *Mytilus edulis* | Med | *Med_Toll-I-1,Med_Toll-I-2,Med_Toll-I-3* |
| *Rapana bezona* | Rbe | *Rbe_Toll-I,Rbe_Toll-2* |
| *Ruditapes philippinarum* | Rph | *Rph_Toll-I,Rph_Toll-2* |
| *Babylonia areolata* | Bar | *Bar_Toll-I,Bar_Toll-2* |
| *Meretrix meretrix* | Mme | *Mme_Toll-I,Mme_Toll-2-1,Mme_Toll-2-2,Mme_Toll-2-3,Mme_Toll-2-4,Mme_Toll-2-5,Mme_Toll-2-6* |
| *Cymbium melo* | Cme | *Cme_Toll-I* |
| *Scapharca subcrenata* | Ssu | *Ssu_Toll_I* |
| *Chlamys farreri* | Cfa | *Cfa_Toll-I-1,Cfa_Toll-I-2,Cfa_Toll-2-1,Cfa_Toll-2-2* |
| *Saxidomus purpuratus* | Spu | *Spu_Toll-I,Spu_Toll-2* |
| *Cyclina sinensis* | Csi | *Csi_Toll-I,Csi_Toll-2* |
| *Tegillarca granosa* | Tgr | *Tgr_Toll-I-1,Tgr_Toll-I-2* |
| *Solen strictus* | Sst | *Sst_Toll-I-1,Sst_Toll-I-2,Sst_Toll-2* |
| *Sinonovacula constrzcta* | Sco | *Sco_Toll-I,Sco_Toll-2* |
| *Hyriopsis cumingii* | Hcu | *Hcu_Toll-I,Hcu_Toll-2-1,Hcu_Toll-2-2* |
| *Crassostrea gigas* | Cgi | *Cgi_Toll-I* |
| *Peronidia zyonoensis* | Pzy | *Pzy_Toll-I-1,Pzy_Toll-I-2,Pzy_Toll-2* |
| *Neptunea cumingi* | Ncu | *Ncu_Toll-2* |
| *Jinqianbei* | Jqb | *Jqb_Toll-I-1,Jqb_Toll-I-2,Jqb_Toll-I-3,Jqb_Toll-2-1,Jqb_Toll-2-2,Jqb_Toll-2-3* |
| *Qicaibei* | Qcb | *Qcb_Toll-I,Qcb_Toll-2* |
| *Wenbei* | Wb | *Wb_Toll-I-1,Wb_Toll-I-2,Wb_Toll-2-1,Wb_Toll-2-2,Wb_Toll-2-3* |
| *Hongbei* | Hb | *Hb_Toll-I* |
