# Supplementary material for: Enhanced antimicrobial peptide-induced activity in the mollusc Toll-2 family through evolution via tandem Toll/interleukin-1 receptor
Source: R Soc Open Sci. 2016 Jun 15;3(6):160123. doi: 10.1098/rsos.160123 (PMC4929906; doi:10.1098/rsos.160123)
Supplement: Primers used in this study [file rsos160123supp3.doc]

**Primers used in this study**

Pac-*Hcu_Toll-2-2*-F: 5'-TATggtaccATGATAAGCGGACTGCAGCATTTAGA-3'

Pac-*Hcu_Toll-2-2*-R1: 5'-TTAgggcccTCTTTCATCGGCAAGGAATACAGAA-3'

Pac-*Hcu_Toll-2-2*-R2: 5'-TTAgggcccTCTGTGATCTCTATCGCAACATCATC-3'

Pac-*Hcu_Toll-I*-F: 5'-TATggtaccATGGTGAACTTCCTAACAGTAAATCT-3'

Pac-*Hcu_Toll-I*-R: 5'-TTAgggcccTCCACGTTTTGAACTTGTACTTTGC-3'

Mve-Toll-I-1-R: 5’-TATTTCTTAGGTCGGTCATCGCCGTGTC-3’

Mve-Toll-I-2-F: 5’-CTGCGATGTACCAGGCCACGGACTAATG-3’

Mve-Toll-I-3-F: 5’-CACAGTTATGCCAAGTATTTCTCACG-3’

Mve-Toll-I-3-R: 5’-GGCAGGATGCTCAACATCAGAAGGTG-3’

Pab-Toll-I-1-F: 5’-CGATTTTGGACTTGGACAGAGGGGC-3’

Pab-Toll-I-1-R: 5’-GCCCCTCTGTCCAAGTCCAAAATCG-3’

Swo-Toll-I-1-R: 5’-GATTGGTGACAAGGCTGCTGTAAGGC-3’

Pru-Toll-I-F: 5’-TGTATTTTTTCTGCAACAGTTCAGCCTTCG-3’

Tte-Toll-I-F: 5’-TGCCATCTCCGACATCACACCCATACAG-3’

Cpl-Toll-I-1-F: 5’-TCAGGAAACTGGAAGGCAAAAAACCGT-3’

Cpl-Toll-I-1-R: 5’-TAGTTTCATAAGGACACCTCTTTGCCGT-3’

Cpl-Toll-I-2-F: 5’-CCCAGGGACAAATACAAGGTGGATGAGC-3’

Cpl-Toll-I-2-R: 5’-CAACTTGCTCATCCACCTTGTATTTGTCCC-3’

Mir-Toll-I-3-R: 5’-CATCACCCGTCCTTTGTTCTTGTCCA-3’

Med-Toll-I-2-F: 5’-GGAATGGGTGAAAACCTGGGCTGACAAC-3’

Med-Toll-I-2-R: 5’-GTTGTCAGCCCAGGTTTTCACCCATTCC-3’

Med-Toll-I-3-F: 5’-ATCGGATTTTGTCATCATATTCAGCGTG-3’

Med-Toll-I-3-R: 5’-ACACCACCGATTGCTGCTGATTGAGG-3’

Mme-Toll-I -F: 5’- ACAACAATCCTGGTGAAGATGAATGCTG-3’

Mme-Toll-I-R: 5’-AAGGTGTCATTACTGATTTCCGTTATTTTA-3’

Cme-Toll-I-R: 5’-GAACAGGTGAGTGTGTGAAGGTCAAGATGC-3’

Jqb-Toll-I-2-F: 5’-TCTGTGACTGTGAGATGTACCCACTTGC-3’

Jqb-Toll-I-2-R: 5’-AGTCGCATTTGAAGGGGTTTCCGTGT-3’

Jqb-Toll-I-3-F: 5’-TTACTGGCATAGACCGCTACACATTCGC-3’

Jqb-Toll-I-3-R: 5’-CCTGGAATGGGTGTTTCAGATTGTGTGT-3’

Ssu-Toll-I-F: 5’-AGCAGCCGTCTGGGCAAAGAAATG-3’

Cfa-Toll-I-1-F: 5’-ACCTGGATGGCGGAATGGATAAAGCG-3’

Cfa-Toll-I-1-R: 5’-AACTGGTGACCGCTTTATCCATTCCGCC-3’

Cfa-Toll-I-2-F: 5’-GAGCCGCAGACCTATCGGGTGTTTTGAG-3’

Cfa-Toll-I-2-R: 5’-GACTCTCCCCGTCATCGTCACAGGTG-3’

Spu-Toll-I-R: 5’-TCCAGTCCACCATCCACAGCATCTCACA-3’

Csi-Toll-I-F: 5’-GTTGATAACGGCGAAAAGATGTCC-3’

Csi-Toll-I-R: 5’-TCTCATAAGGACATCTTTTCGCCG-3’

Pab-Toll-I-2-R: 5’-CGTCCGTGACTGTGTTCCAATGTTTCCG-3’

Tgr-Toll-I-1-R: 5’-CGACACCAATGACGACGGCTAACAAAGA-3’

Sst-Toll-I-1-F: 5’-CTCTCATTGTAACAGCCAAAAGATGTCCGT-3’

Sst-Toll-I-1-R: 5’-TGAGTCAACCGCATACTTATCCGCCG-3’

Sst-Toll-I-2-F: 5’-TCTGCTGCTTTACTGAAGAACACGCTCG-3’

Sst-Toll-I-2-R: 5’-GTGTTTCAACCGTAACCTCGGCTATT-3’

Sco-Toll-I-F: 5’-CGGGCTATTATGATTGAGTCCAGCAGGC-3’

Sco-Toll-I-R: 5’-ACAGTCTCTCGCTGGCAGGGCAACAC-3’

Cgi-Toll-I-F: 5’-CGCCCTTGTCATCGTGGCTGTTATTTGG-3’

Pzy-Toll-I-1-F: 5’-ACATCCTTTCAGAGAACTGCTGCGTG-3’

Pzy-Toll-I-2-F: 5’-CTCTACCCGACTATGTGCCATCATCCAA-3’

Pzy-Toll-I-2-R: 5’-TCCAAGAAGCGTTTACCCTGCCTCCG-3’

Qcb-Toll-I-F: 5’-GGTGTCTACGCAAGAGATGGTTCCGCTG-3’

Qcb-Toll-I-R: 5’-TTCGTTTCGTATGGACATCGTTTGGCGG-3’

Hb-Toll-I-F: 5’-TCGCCAATGGGTATTAAAAGAGTTGGTG-3’

Hb-Toll-I-R: 5’-CACCAACTCTTTTAATACCCATTGGCGA-3

Cpl-Toll-2-2-F: 5’-TCATTCTGGGATGCTGACCGACTGGG-3’

Cpl-Toll-2-3-F: 5’-AGACCAACTATCTGATCGTCATCGTGCG-3’

Cpl-Toll-2-3-R: 5’-CACGCACGATGACGATCAGATAGTTG-3’

Cpl-Toll-2-4-R: 5’-GGGAATAAAGTCTCGGTCAGGGAGGCAC-3’

Cpl-Toll-2-5-F: 5’-GCGACATGGCTCGTGATTTTGTCATAGG-3’

Cpl-Toll-2-5-R: 5’-TGCTGTTGCGTGTTTCTATTGATGTG-3’

Mme-Toll-2-1-R: 5’-CCAGTGGGAAGGCATTTTGGTTTAC-3’

Mme-Toll-2-4-F: 5’-ACCAGCGAGCAGTTGCGGGACAAATGAA-3’

Mme-Toll-2-4-R: 5’-TTCATTTGTCCCGCAACTGCTCGCTG-3’

Mme-Toll-2-5-F: 5’-TGATGCCAAAGACAGGAATGGATAGTGC-3’

Mme-Toll-2-5-R: 5’-GGACATCACCGTGGTCAGTATAAACATCTT-3’

Mme-Toll-2-6-F: 5’-ATCAGAAGATTGGAAAACGTCGTCTGACAG-3’

Mme-Toll-2-6-R: 5’-GTCCTTAGAATCAACAAACCTCCCGTGC-3’

Jqb-Toll-2-1-R1: 5’-CGGAAGTGTGACAAGTGGGTTATGATGA-3’

Jqb-Toll-2-1-R2: 5’-CCTGAAATACACCGTCTGGCAAATC-3’

Jqb-Toll-2-2-F: 5’-GTCAGTCGGATACATGATCGTTTACC-3’

Jqb-Toll-2-2-R: 5’-ATTGAACAAACTGCGTTGGCACCTCGGG-3’

Jqb-Toll-2-3-F: 5’-GAGTTTTTGCCAGGCAGTTCCAAAGGTG-3’

Jqb-Toll-2-3-R: 5’-TCCTTCATCAAGCTCGCACGATTGTC-3’

Cfa-Toll-2-1-R: 5’-TAGATTCAGCGTGTCTGTCAGTCGGTAG-3’

Cfa-Toll-2-2-F: 5’-ACAGCGTTCACGGCGAGACAAATAC-3’

Cfa-Toll-2-2-R: 5’-ATACAAAGAGGGGGTCGTAAACAGCGTC-3’

Spu –Toll-2-R: 5’-GTAAACCCCTTTGTGTCAAGAAGTGGCA-3’

Spu –Toll-2-F: 5’-GAGATGCGATACGTCGTTATGCCATCAG-3’

Csi-Toll-2-F: 5’-GTGTACTTTGGTGTCCATCCATTTGACAGG-3’

Csi-Toll-2-R: 5’-CAGACTTATCTTATGTAGTGTTCCGTGG-3’

Pab -Toll-2-1-F: 5’-TGCCCGATAAGCAAAGCACCTGTCAA-3’

Pab-Toll-2-1-R: 5’-GATGTTGACAGGTGCTTTGCTTATCGGG-3’

Pab -Toll-2-2-F: 5’-CGGAGTGGGTTATGTCTCAGATTGTTGG-3’

Pab -Toll-2-2-R: 5’-CCAGTCATTGGATACACAGAAAAGCATT-3’

Ncu-Toll-2-F: 5’-ACGTCAGCACGGAGGACAAGCACTTC-3’

Ncu-Toll-2-R: 5’-CACCAGCAGCTCGTTCCTCTCCCAGG-3’

Sst -Toll-2-F: 5’-CTTCCTCAGATGGATTTGCTGTGGCGTG-3’

Sst -Toll-2-R: 5’-GCCCCACGCCACAGCAAATCCATC-3’

Sco-Toll-2-R: 5’-CCACATTCGCAGTTGACACTTCTGGACACG-3’

Sco-Toll-2-F: 5’-CGCTGACGAAGACGAGGATTACG-3’

Hcu-Toll-2-2-F: 5’-TGCCGCACTCTTTGGATGAAGGATTGGATG-3’

Hcu-Toll-2-2-R: 5’-AGGATGATTGTACTTGTAACGGTTGGGATG-3’

Pzy-Toll-2-1-F: 5’-TTCTGGAGGAGCAAGGGTTCCGATT-3’

Pzy-Toll-2-1-R: 5’-ATCATTGACTTCCCGTTGAAAGCAGGCG-3’

Wb-Toll-2-1-R: 5’-GCTGCTCGCTGAATCAATGACGCTAT-3’

Wb-Toll-2-2-F: 5’-GCTGATGACACTCCTGAAGCAGAAACGA-3’

Wb-Toll-2-2-R: 5’-CAGCACCCAATGGGAATTCCATATCCAG-3’

Wb-Toll-2-3-F: 5’-AGGACAACACCTGTAACAGACTGGAGCC-3’

Wb-Toll-2-3-R: 5’-ACTTGGCTCCAGTCTGTTACAGGTGTTG-3’

Qcb-Toll-2-R: 5’-TCTCGGAGGATGTAGACCAGTCGTTTGA-3’
