# Supplementary figures and images for: Enhanced antimicrobial peptide-induced activity in the mollusc Toll-2 family through evolution via tandem Toll/interleukin-1 receptor
Source: R Soc Open Sci. 2016 Jun 15;3(6):160123. doi: 10.1098/rsos.160123 (PMC4929906; doi:10.1098/rsos.160123)

Supplementary material, S4. Detail of five motifs.

Motif 1


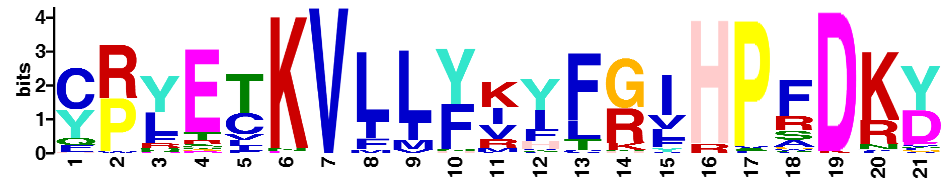


Motif 2


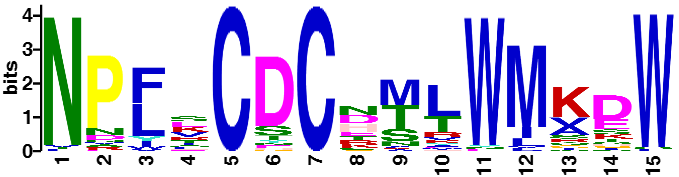


Motif 3


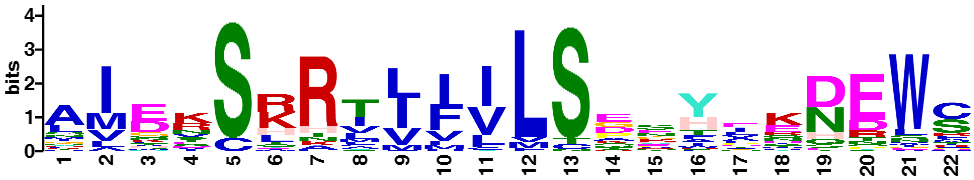


Motif 4


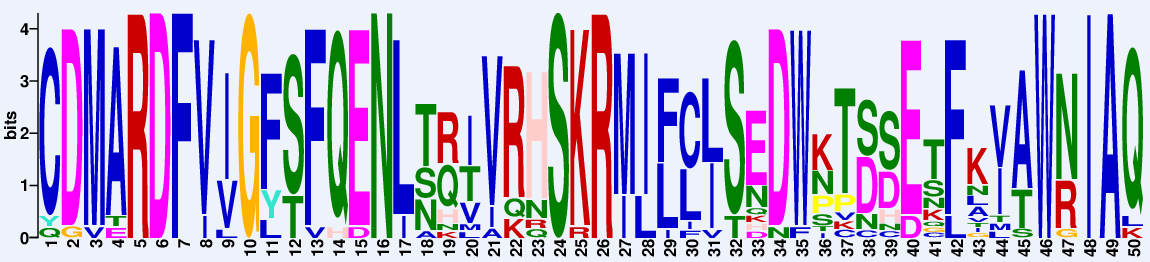


Motif 5


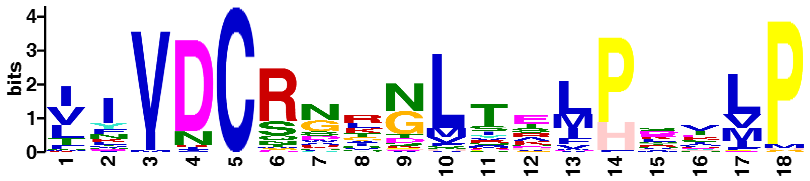

Supplement: Supplementary material, S4. Detail of five motifs. [file rsos160123supp4.doc]
